# Psychological rehabilitation for isolated patients with COVID-19 infection: A randomized controlled study

**DOI:** 10.1371/journal.pone.0278475

**Published:** 2022-12-27

**Authors:** Jae Hyu Jung, Jong Jin Won, Jin Young Ko

**Affiliations:** 1 Department of Occupational Therapy, Gyeonggi Provincial Medical Center, Suwon, Korea; 2 Department of Local Public Health Care Headquarters, Gyeonggi Provincial Medical Center, Suwon, Korea; 3 Department of Rehabilitation, Seoul National University Bundang Hospital, Seongnam, Korea; Prince Sattam Bin Abdulaziz University, College of Applied Medical Sciences, SAUDI ARABIA

## Abstract

**Objective:**

To improve the mental health of isolated patients with COVID-19 by face-to-face psychological rehabilitation program.

**Design:**

Randomized controlled study.

**Setting:**

Single community-based hospital.

**Participants:**

109 patients (52 in experimental group, 57 in control group) with COVID-19 were recruited from May 27 to September 17, 2021.

**Interventions:**

A psychological rehabilitation program that consists of education, craft, and physical activity. The activity program was provided by a multidisciplinary rehabilitation team of doctors, nurses, occupational therapists, and physical therapists. The purpose of the education was to provide accurate information about COVID-19, and craft and physical activity were for improving physical health, occupational balance, participation in activities, and reducing boredom.

**Main outcome measures:**

The primary outcome was degree of anxiety assessed using the Zung Self-Rating Anxiety Scale (SAS). Secondary outcomes were severity of depression, and quality of sleep assessed using the Zung Self-Rating Depression Scale (SDS), Patient Health Questionnaire-9 (PHQ-9), Visual Analysis Scale (VAS), and the Korean version of the Insomnia Severity Index (ISI-K).

**Results:**

Isolated patients complained of anxiety, depression, and insomnia the most in the early stages of hospitalization and isolation. In addition, the psychological rehabilitation program significantly improved mental health scale, including scores of SAS (*F* = 12.46, *p* = .001), SDS (*F* = 6.76, *p* = .01), and ISI-K (*F* = 4.41, *p* = .04).

**Conclusions:**

The psychological rehabilitation program is effective for improving anxiety, depression, and quality of sleep for isolated patients with COVID-19.

## Introduction

The COVID-19 pandemic is known to have caused mental health problems among the general population [1] and healthcare providers [2]. Although the mental health problems of hospitalized COVID-19 patients have not been studied in depth, it is known that COVID-19 patients suffer from psychological problems such as depression, anxiety and sleep disturbances due to hospitalization and isolation [3,4].

Due to the fear of physical contact and lack of protective materials, face-to-face interaction was limited. Although some kinds of telecommunication was done in the case of isolated patients [5,6], there are several barriers such as technical difficulties, limited familiarity with telemedicine, and loss of non-verbal communication that normally occurs in face-to-face interactions [7,8]. Owing to these barriers and depending on the social environment, patients may not be able to fully express their emotional and mental health problems [9].

The main objective of the study, therefore, was to measure the effects of newly developed face-to-face psychological rehabilitation program among isolated patients with COVID-19.

## Method

### Design and participants

**Fig 1:**
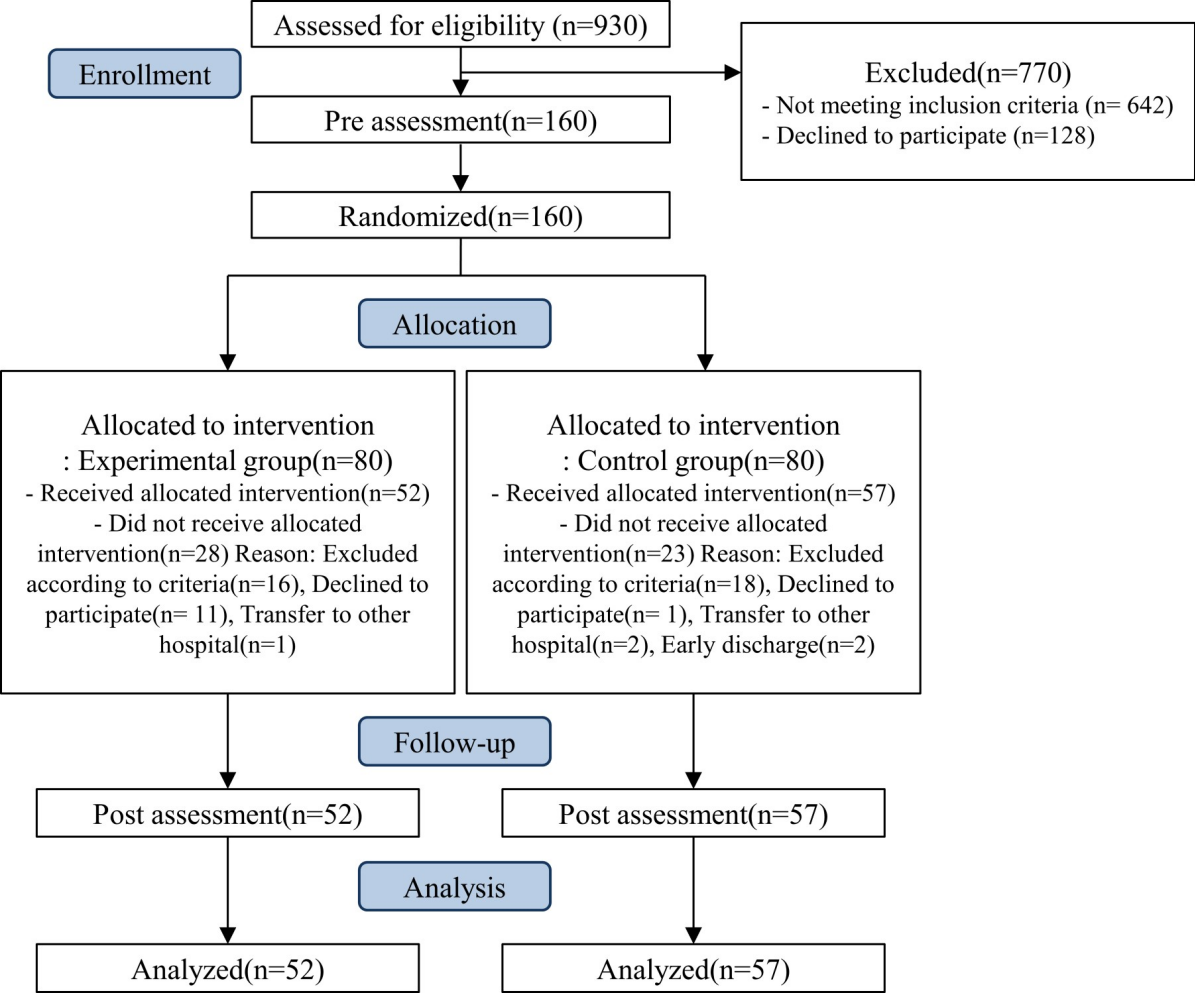
CONSORT flow diagram of recruitment, allocation, and participation of study.

A total of 160 patients with confirmed COVID-19, admitted to a single community-based hospital from May 27 to September 17, 2021, were included in the study (Fig 1). A priori analysis was conducted by G Power 3.1 software (f = 0.25, α = 0.05, 1-β = 0.95, ANOVA: repeated measures), and the results were calculated to show a sample size of 86. We tried to recruit a minimum of 108 subjects to account for a dropout rate of 20%. The study was also approved by the IRB as minimal risk, and enrollment continued for the duration of the study. The patients were randomly divided into experimental and control groups according to their bed number. Block randomization method was used and block size were 6, 8, 10, 12, and 14. The patients were recruited according to the following inclusion criteria: (1) diagnosed as COVID-19 positive as per the polymerase chain reaction (PCR) test; (2) oxygen saturation of 95% or higher and hemodynamically stable; (3) admitted to the isolation ward; (4) no history of psychiatric diagnosis; (5) at least 18 years of age; (6) able to understand the purpose of the research and consent. Participants were excluded if they: (1) were not medically stable; (2) had difficulty using Korean or other communication problems. (3) had a history of serious, diagnosed psychiatric disease: depression, anxiety disorder, insomnia, bipolar disorder, and schizophrenia. Subjects in this study were part of a clinical trial(KCT0007222). The study protocol was approved by the institutional review board of Seoul National University Bundang Hospital(B-2104-678-301), which waived written informed consent due to the risk of virus transmission. The research team provided information regarding the aim of the study and received online consent by clicking “I agree” button from all participants before starting the study.

### Outcome measure

The Zung Self-Rating Anxiety Scale (SAS) [10] and Visual Analysis Scale (VAS) were used to assess the level of anxiety, and the Zung Self-Rating Depression Scale (SDS), [11] Patient Health Questionnaire-9 (PHQ-09), [12] and Visual Analysis Scale (VAS) were used to evaluate the degree of depression. Also, to assess the quality of sleep, we used the Korean version of the Insomnia Severity Index(ISI-K) [13]. The SAS and SDS contain 20 items each, rated on a 4-point Likert scale ranging from 1 (a little of the time) to 4 (most of the time). The SAS is divided into four levels: no anxiety (≤44), mild to moderate (45–59), severe (60–74), and extremely severe (≥75). The SDS is divided into four levels: no depression (≤49), mild to moderate (50–59), severe (60–69), and extremely severe (≥70). The PHQ-9 has nine questions rated on a 4-point Likert scale ranging from 0 (not at all) to 3 (nearly every day). It is divided into four levels: no depression (≤4), mild (5–9), moderate (10–19), and severe (≥20). The ISI–K has seven questions rated on a 5-point Likert scale ranging from 0 (no problem) to 4 (very severe). It is divided into four categories: absence of insomnia (≤7), sub-threshold mild (8–14), moderate (15–21), and severe (≥22). Participants completed the outcome measurement at the initial and post-interventions.

### Intervention protocol

**Fig 2:**
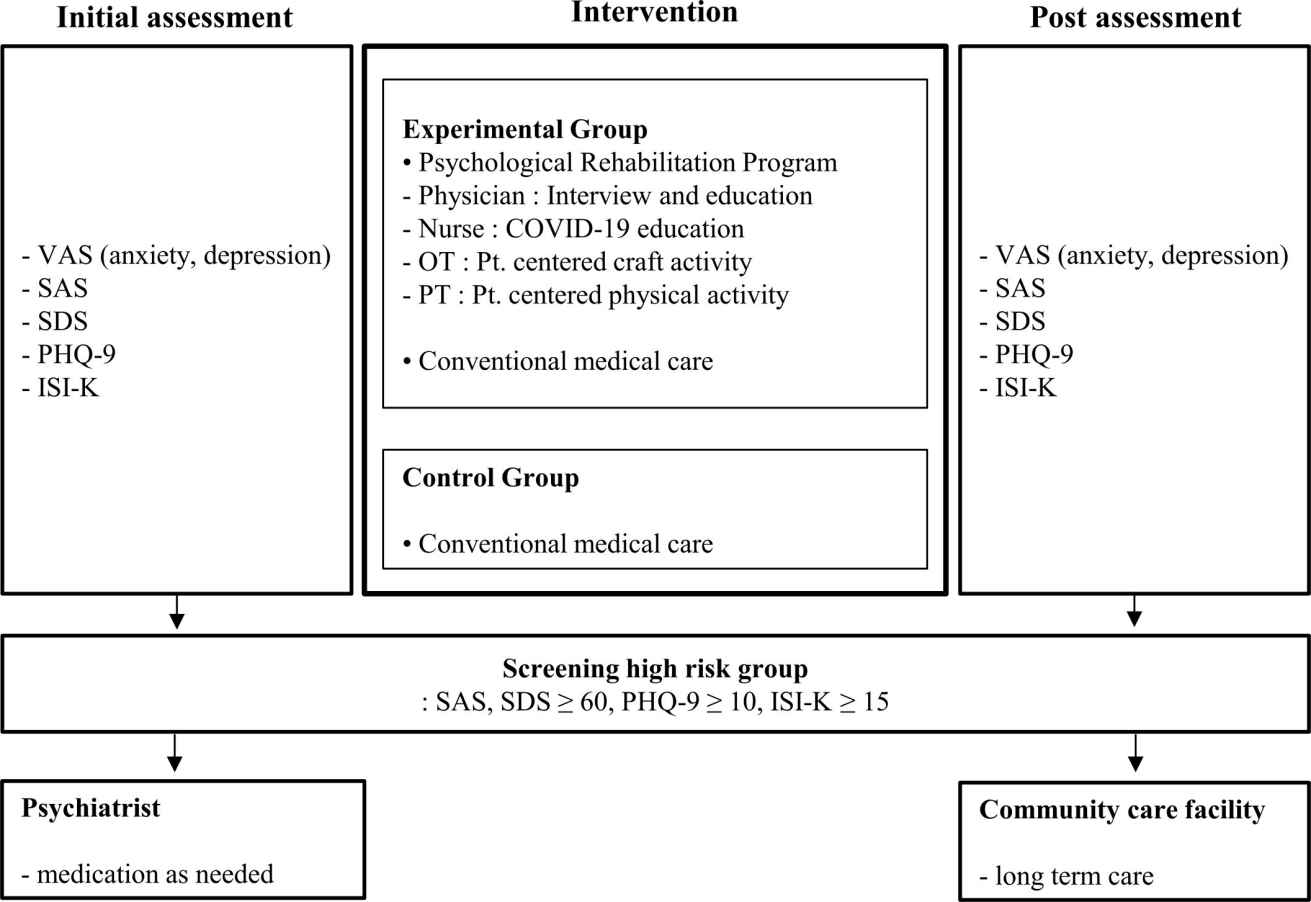
Intervention protocol: A psychological rehabilitation program consisting of COVID-19 education, crafts, and physical activities.

Fig 2 displays the protocols for the intervention used in this study. Both the experimental and the control group were screened for mental health risk, symptomatic treatment for COVID-19-related symptoms, and antiviral therapy. A psychological rehabilitation program, comprising COVID-19 education, craft, and physical activities was conducted only for the experimental group. The purpose of the COVID-19 education, imparted by a nurse, was to debunk misinformation about COVID-19 and to provide accurate information about it. The education included epidemiologic characteristics, symptoms, treatment methods, prognosis, and precautions against COVID-19 [14,15]. A 20-minute session was conducted after the pre-evaluation. Next, a craft activity was organized by an occupational therapist. The purpose of this activity was occupational balance, participation in activities, and reducing boredom during hospitalization. The procedure was as follows: Step 1. A list of possible craft activities that could be done in the isolation ward was provided. An occupational therapist visited the isolation ward to discuss the activity options with the patient. Step 2. An activity was selected according to the patient’s preference gauged through an interview conducted by the occupational therapist. Craft activities included knitting, jewel cross-stitching, coloring, block-making, etc. The craft activity list was composed of a wide variety taking into consideration factors such as age, gender, nature of disease, and the facilities available at the program venue. Step 3. The occupational therapist visited the isolation room and provided emotional support while observing and conversing with the patient during the selected activity. Conversations mainly centered around the hospital stay. The craft activity was provided for 8 days, 20 mins each day. Next, the patients trained in exercises guided by a physical therapist, for 8 days, 20 mins each day. Physical activities included stretching, strength training, and breathing exercises. For stretching, neck stretch, side stretch, chest stretch, hip stretch, and waist stretch were performed. Strength training consisted of wall push-ups, squats, and heel raisers. Breathing exercises included diaphragmatic breathing, pursed-lip breathing technique, and square box breathing. The activity started as an exercise for improving joint range of motion and gradually progressed to exercise for strengthening. The patient’s symptoms such as muscle pain and breathing difficulties were observed. All physical activity was performed with reference to modules 3 and 4 of the WHO COVID-19 rehabilitation guidelines, and all psychological rehabilitation programs were conducted in the patient’s isolation ward. For protection against the virus, the therapist, carried out the intervention wearing a powered aid purifying respirator [16].

Patients were screened for high-risk mental health conditions. Psychiatric linkage was performed on patients who were evaluated as high-risk in the initial evaluation. This was done for members of both the experimental and control groups. The range of the high-risk group was set at 60 points or higher for SAS, SDS, 10 points or higher for PHQ-9, and 15 points or higher for ISI-K [17,18]. If the patient declined the service, the connection was not made, and the researcher continuously monitored levels of anxiety, depression, and insomnia for signs of deterioration during the interviews. Patients evaluated as high-risk groups in the discharge evaluation were referred to a community mental health institution to receive medical care, after obtaining their consent.

### Statistical analysis

Data analysis was performed using SAS statistical software version 9.4 (SAS Institute, Cary, NC). To compare descriptive data of the two groups, we used independent t-test, chi-square and Fisher’s exact test.

The repeated measures ANOVA was used to measure changes over time between groups for the pre-post repeated measures of VAS, SAS, SDS, PHQ-9, ISI-K. The repeated covariance type was used for unstructured, and fixed effects included group (experimental and control group) and time (pre- and post-intervention). The difference between the two groups over time was analyzed by assessing the interaction between the groups using the measurement time points. The level of statistical significance was set at P < 0.05.

## Results

### Participant flow

A total of 160 patients with COVID-19 were recruited from May 27 to September 17, 2021. They were randomly allocated to the experimental (n = 80) or control group (n = 80). During the program, 28 patients in the experimental group and 23 patients in the control group were excluded. In the experimental group, 16 patients had decreased oxygen saturation (90–95%), 11 patients declined program, and 1 patient was transferred to another hospital. In the control group, 18 patients had decreased oxygen saturation (90–95%), 1 patient declined the program, 2 patients were transferred to other hospitals, and 2 patients were discharged early. In total, 109 patients (52 patients in the experimental group, 57 patients in the control group) completed the trial (Fig 1).

### Patient demographics

Table 1 summarizes the demographic and clinical characteristics of the participants. The experimental group consisted of 28 males and 24 females, with an mean age of 51.06±16.42 years and mean isolation duration of 13.54±3.27days. The control group consisted of 24 males and 44 females, with an mean age of 45.96±17.20 years and mean isolation duration of 14.00±4.18 days. HTN and dyslipidemia were the most common underlying diseases in both groups. Additionally, the most common clinical symptoms were pneumonia, myalgia, cough & sputum, headache, and sore throat in both groups. No significant difference was found between the groups in demographic and clinical characteristics (p>.05).

**Table 1:** Demographic and clinical characteristics according to groups.

Classification			EG (N = 52)	CG (N = 57)	*p*
Gender, n (%)	Male		28(53.85%)	24(42.11%)	.22
	Female		24(46.15%)	44(57.89%)	
Age (year), M (SD)			51.06(16.42)	45.96(17.20)	.74
Duration of isolation (day), M (SD)			13.54(3.27)	14.00(4.18)	.07
Underlying disease, n (%)		HTN	17(32.69%)	17(29.82%)	.75
		DM	7(13.46%)	6(10.53%)	.64
		Dyslipidemia	15(28.85%)	12(21.05%)	.35
		CVD	0(0.00%)	1(1.75%)	^+^.52
		CAD	1(1.92%)	0(0.00%)	^+^.48
		Etc	14(26.92%)	10(17.54%)	.24
Vaccinated before infection			18(34.62%)	12(21.05%)	.11
Clinical symptoms, n (%)		Fever (over 37.5°C)	36(69.23%)	37(64.91%)	.63
		Pneumonia	37(71.15%)	39(68.42%)	.76
		Myalgia	24(46.15%)	23(40.35%)	.54
		Chilling	14(26.92%)	22(38.60%)	.20
		Cough & sputum	37(71.15%)	45(78.95%)	.35
		Fatigue	5(9.62%)	2(3.51%)	.19
		Headache	20(38.46%)	29(50.88%)	.19
		Sore throat	22(42.31%)	29(50.88%)	.37
		Nausea & vomiting	7(13.46%)	12(21.05%)	.30
		Diarrhea	3(5.77%)	5(8.77%)	^+^.24
		Dyspnea	7(13.46%)	15(26.32%)	.09
		Hyposmia & hypogeusia	9(17.31%)	8(14.04%)	.64
		Dizziness	4(7.69%)	6(10.53%)	.61
		Asymptomatic	1(1.92%)	1(1.75%)	^+^.50

EG = Experimental group; CG = Control group, M = Mean, SD = Standard deviation, HTN = Hypertension, DM = Diabetes, CVD = Cerebrovascular disease, CAD = Coronary artery disease.

^+^: Fisher`s exact test.

Duration of isolation means the period from the start date of quarantine to the date of discharge evaluation.

### Anxiety

**Fig 3:**
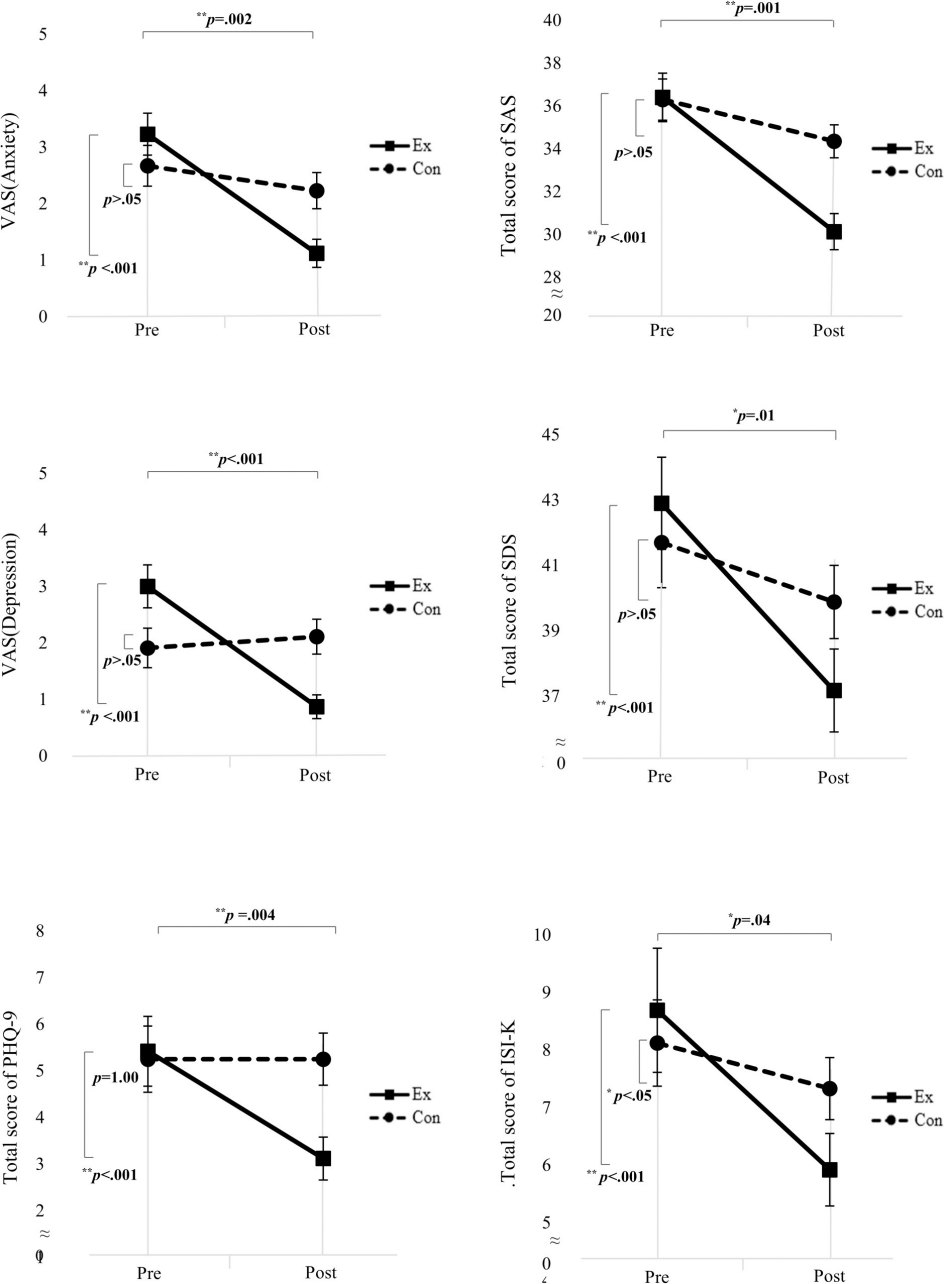
Patterns of change in mental health conditions over a experimental period.

The VAS of anxiety (*F* = 10.46, *p* = .002) and the total score of SAS (*F* = 12.46, *p* = .001) significantly decreased in the experimental group compared with the control group in the time × group analysis (Table 2, Fig 3). The VAS of anxiety (*F* = 24.29, *p* < .001) and SAS total score (*F* = 45.04, *p* < .001) significantly decreased over time in both groups.

**Table 2:** Comparison of changes in variables within and between groups.

Variable		Pre	Post	*p*	Time		Time x group Interaction	
		M (SD)	M (SD)		*F*	*p*	*F*	*p*
VAS (Anxiety)		EG	3.23(2.67)	1.12(1.78)	< .001**	24.29	< .001**	10.46	.002**
		CG	2.67(2.75)	2.23(2.40)	.23				
SAS		EG	36.40(8.18)	30.13(6.08)	< .001**	45.04	< .001**	12.46	.001**
		CG	36.30(7.27)	34.35(5.83)	.03				
VAS (Depression)		EG	3.00(2.74)	0.87(1.50)	< .001**	16.30	< .001**	23.42	< .001**
		CG	1.91(2.65)	2.11(2.32)	.56				
SDS		EG	42.90(10.18)	37.17(9.16)	< .001**	25.30	< .001**	6.76	.01*
		CG	41.70(8.59)	39.88(8.49)	.08				
PHQ-9		EG	5.42(5.43)	3.12(3.30)	< .001**	8.58	.004**	8.58	.004**
		CG	5.25(5.33)	5.25(4.23)	1.00				
ISI-K		EG	8.69(7.77)	5.92(4.57)	.001**	14.25	< .001**	4.41	.04*
		CG	8.12(5.69)	7.33(4.15)	.14				

EG = Experimental group; CG = Control group; M = Mean; SD = Standard deviation. SAS = Zung Self-rating Anxiety Scale; SDS = Zung Self-rating Depression Scale; PHQ-9 = Patient Health Questionnaire-9; ISI-K = Korean version of Insomnia Severity Index.

*p < .05

**p < .01.

When analyzing within-group changes, the VAS of anxiety (*p* < .001) and total score of SAS (*p* < .001) in the experimental group significantly decreased. In the control group, the VAS of anxiety (*p* = .23) and total score of SAS (*p* = .03) decreased.

### Depression

In the time × group analysis, the VAS of depression (*F* = 23.42, *p* < .001), total score of SDS (*F* = 6.76, *p* = .01), and PHQ-9 (*F* = 8.58, *p* = .004) significantly decreased in the experimental group compared with the control group (Table 2, Fig 3). The VAS of depression (*F* = 16.30, *p* < .001), SDS (*F* = 25.30, *p* < .001), PHQ-9 total score (*F* = 8.58, *p* = .004) significantly decreased over time in both groups.

When analyzing within-group changes, the VAS of depression (*p* < .001), total score of SDS (*p* < .001), and PHQ-9 (*p* < .001) significantly decreased in the experimental group. In the control group, the VAS of depression (*p* = .56), total score of SDS (*p* = .08), and PHQ-9 (*p* = 1.00) decreased, but not significantly.

### Insomnia

In the time × group analysis, the total score of ISI-K (*F* = 4.41, *p* = .04) significantly decreased in the experimental group compared with the control group (Table 2, Fig 3). ISI-K total score significantly decreased with time in both groups.

The ISI-K total score in the experimental group decreased from 8.69±7.77 to 5.92±4.57 after the intervention (*p* < .001). In the control group, ISI-K total score decreased from 8.12±5.69 to 7.33±4.15(*p* = .14) post- intervention.

## Discussion

To our knowledge, this is one of the first studies on psychological rehabilitation for COVID-19 patients admitted to the hospital isolation ward. According to our results, patients complained of anxiety, depression, and insomnia the most in the early stages of hospitalization and isolation. In addition, psychological rehabilitation programs reduced mental health problems such as anxiety, depression, and insomnia.

Previous research has shown that patients isolated from COVID-19 are vulnerable to mental illness, including anxiety, and experience social guilt about infecting others[19]. And also hospitalization and isolation are known to cause physical inactivity, boredom, sleep deprivation, and stress [3,20]. In particular, boredom is known to cause psychological problems such as depression, aggression, and suicidality [21].

The effect of psychological rehabilitation on mental illness can be explained by the effects of education, physical activity, and craft activity, which are sub-items of psychological rehabilitation. There have been prior studies on physical exercise and activity participation. Exercise and body relaxation therapy was observed to improve the mental health of isolated people [22–24]. Participation in activities is known to relieve boredom [25], and daytime activities are effective to improve sleep quality [26]. However, the most of studies were conducted on incarcerated individuals [22,27] or self-isolated rather than being isolated in hospitals or treatment centers [23]. Therefore, this study has clinical significance in that it targeted patients isolated by COVID-19 infection.

In addition, due to strict adherence to quarantine rules, therapists’ fear of being infected with COVID-19 was not observed at all during the intervention. Researchers did not complain of COVID-19-related symptoms during the intervention, nor were they diagnosed with COVID-19. Exercise, education, and participation in activities are considered to have improved the mental health of patients in an environment that guarantees the safety of medical staff and therapists as part of a complex program.

This study has several limitations. First, the results of this study may not reflect the information of all patients depending on the acceptance rate and exclusion rate. This may result in a selection bias affecting the validity and generalizability of result. Second, there were no baseline assessments of the patients’ anxiety and depression levels prior to admission, so it is possible that they were already high due to the pandemic, whether hospitalized or not. Third, although our findings may be generalized to Korean patients, they may not be applicable to other countries. Additionally, we did not evaluate the patients’ conditions after discharge. Therefore, it was not possible to determine whether their mental health changed post-discharge. Further research, including patient follow-up, is required.

## Conclusion

According to the results of this study, among COVID-19 patients who were hospitalized, mental health deterioration occurred, and a psychological rehabilitation program was required to actively prevent and treat it. Based on these findings, it is recommended that isolated patients with COVID-19 should undergo a psychological rehabilitation program.

## Supporting information

S1 Checklist CONSORT 2010 checklist of information to include when reporting a randomised trial*. (DOCX)

S1 File (PDF)

S2 File (PDF)
