# Supplementary material for: Psychological rehabilitation for isolated patients with COVID-19 infection: A randomized controlled study
Source: PLoS One. 2022 Dec 27;17(12):e0278475. doi: 10.1371/journal.pone.0278475 (PMC9794049; doi:10.1371/journal.pone.0278475)
Supplement: S1 File — (PDF) [file pone.0278475.s002.PDF]

Status : Approved
First Submitted Date : 2022/04/13 Registered Date : 2022/04/25
Last Updated Date : 2022/04/13

1. Background

CRIS Registration Number	KCT0007222
Unique Protocol ID	B-2104-678-301
Public/Brief Title	psychiatric rehabilitation on COVID-19
Scientific Title	Effect of psychiatric rehabilitation on COVID-19 patients.
Acronym	EPRCP
MFDS Regulated Study	No
IND/IDE Protocol	No
Registered at Other Registry	No
Healthcare Benefit Approval Status	Not applicable

2. Institutional Review Board / Ethics Committee

Board Approval Status	Submitted approval
Board Approval Number	B-2104-678-301
Approval Date	2021-05-26
Institutional Review Board Name	Seoul National University Bundang Hospital Institutional Review Board
Institutional Review Board Address	82, Gumi-ro 173beon-gil, Bundang-gu, Seongnam-si, Gyeonggi-do
Institutional Review Board Telephone	031-787-8801

Data Monitoring Committee	No

3. Contact Details

- Contact Person for Principal Investigator / Scientific Queries

Name	Jin Young Ko
Title	Rehabilitation Physician
Telephone	+82-31-8046-5381
Affiliation	Seoul National University Bundang Hospital
Address	82, Gumi-ro 173beon-gil, Bundang-gu, Seongnam-si, Gyeonggi-do, Republic of Korea

- Contact Person for Public Queries

Name	Jae Hyu Jung
Title	Occupational therapist
Telephone	+82-31-8046-5380
Affiliation	Gyeonggi Provincial Medical Center Ansung Hospital
Address	95, Nampa-ro, Anseong-si, Gyeonggi-do, Republic of Korea

- Contact Person for Updating Information

Name	Jae Hyu Jung
Title	Occupational therapist
Telephone	+82-31-8046-5380
Affiliation	Gyeonggi Provincial Medical Center Ansung Hospital
Address	95, Nampa-ro, Anseong-si, Gyeonggi-do, Republic of Korea

4. Status

Study Site	Single
Overall Recruitment Status	Completed

Date of First Enrollment	2021-05-26 Actual
Target Number of Participant	200
Primary Completion Date	2021-09-17 , Actual
Study Completion Date	2021-09-17 , Actual

- Recruitment Status by Participating Study Site 1

Name of Study	Gyeonggi Provincial Medical Center Ansung Hospital
Recruitment Status	Completed
Date of First Enrollment	2021-05-26 ,

5. Source of Monetary / Material Support

- 1. Source of Monetary/Material Support

Organization Name	Ministry of Health & Welfare
Organization Type	Government
Project ID	2022-ACHCC-26

6. Sponsor Organization

- 1. Sponsor Organization

Organization Name	Seoul National University Bundang Hospital
Organization Type	Medical Institute

7. Study Summary

Lay Summary	- The World Health Organization (WHO) declared a 'Public Health Emergency of International Concern (PHEIC)' on January 30, 2020 due to the worldwide spread of Corona 19. Strict measures are being taken around the world to isolate patients diagnosed or suspected of having COVID-19. In Korea, the cumulative number of confirmed cases, from 30 in early February and 1,000 at the end of February, rapidly increased to 10,000 at the end of April, and as a prolonged national emergency, patients with mild COVID-19 are isolated at a hospital or co

	mmunity treatment center.<br>- Isolation changes daily routines along with social isolation to experience loneliness, excessive worry, and stress.<br>These symptoms are related to mental health, and the experience of disasters also complains of stress responses over time, such as anxiety and depression. This leads to post-traumatic stress disorder, which has a significant impact on mental health. It is reported that 70.8% of confirmed MERS patients suffered psychiatric problems such as depression, insomnia, tension, aggression, memory loss, and auditory hallucinations. Complains of difficulties in communication, feelings of guilt about the contact, and anxiety and depression related to uncertainty.<br>- Patients hospitalized in isolation wards due to COVID-19 wanted to communicate with the outside, understand the disease, and provide entertainment that can cope with boredom. It would be helpful to patient that education, effective communication, closely monitoring, and early intervention. There are many studies that report psychological symptoms in patients diagnosed with COVID-19, but there is still a lack of interventional studies.<br>- COVID-19 patients experience daily changes and reduced activity levels, which can lead to depression, anxiety, and decreased function. In addition, from a psychiatric point of view, the COVID-19 pandemic is reported to cause moral injury. These negative emotions are often observed in isolated patients, which leads to psychological atrophy and devastation. Despite these circumstances, interventions to reduce stress, alleviate negative reactions, and promote physical and mental recovery have not been actively implemented.<br>-The purpose of this study to improve the mental health of isolated patients with COVID-19 by face-to-face psychological rehabilitation program.

8. Study Design

Study Type	Interventional Study
Study Purpose	Supportive Care
Phase	Not applicable
Intervention Model	Factorial
Blinding/Masking	Single
Blinded Subject	Subject
Allocation	RCT
Intervention Type	Behavioral
Intervention Description	1. Physical activity and Breathing Exercises<br>1) Physical activity<br>- Stretching and strengthening exercise<br>- Starting with the joint range of motion, gradually progresses with muscle strengthening movement.<br><br>2) Breathing Exercises and Stabilization Techniques<br>: Exercise is performed twice a day during hospitalization in the following order.

	In the morning, exercise with the researcher(15 minutes), and the patient do exercise on their own at the afternoon(15 minutes).<br><br>(1) Diaphragmatic breathing (3 minutes)<br>① In a lying position, place one hand on your chest and the other on your stomach.<br>② Take a deep breath through your nose to inflate your stomach. At this time, make sure that you do not move your chest as much as possible with your hand on top of your chest.<br>③ Close your mouth and exhale slowly. At this time, the chest should not move as much as possible, and only the movement of the stomach should appear.<br><br>(2) Pursed-lip breathing (3 minutes)<br>① Relax your neck and shoulder muscles.<br>② Inhalation: Inhale slowly through the nose for 2 counts<br>③ Exhalation: Make the shape of your lips like a whistle and exhale slowly for 4 counts<br><br>(3) Square box breathing (3 minutes)<br>① Inhale: Close your eyes. Breathe in through your nose whilst counting to 4.<br>② Hold: Keep the air inside and count to 4.<br>③ Exhale: Breathe out slowly for count of 4. Repeat.<br>④ Hold:Wait another count of 4 until you breathe in through your nose again.<br><br>(4) huff coughing (3 minutes)<br>① Take a seated position.<br>② Breathe in through your mouth. At this point, breathe in a little deeper than usual.<br>③ Activate the abdominal muscles to quickly exhale and exhale 3 times with 'ha', 'ha', and 'ha' sounds.<br><br>(5) Progressive muscle relaxation (3 minutes)<br>① Get into a comfortable position.<br>② Take a few deep breaths and tense up the muscles.<br>③ Exhale and release tension.<br>Upper extremity, shoulder, head, neck, chest, abdomen, lower extremities in order<br><br>2. Occupational activity (Craft activity)<br>- Knitting, curling book, figure making, masterpiece puzzle, etc.<br>- After selecting the activity, the patient wants through a phone interview, the therapist visits and provides it<br>- Educate yourself so that you can do it yourself and check if it was implemented over the phone<br><br>3. Medical and conservative treatment<br>- Isolation and conservative treatment according to COVID-19 response guidelines<br>- Implemented according to the patient's condition by medical staff
Number of Arms	2

Arm 1	Arm Label	Psychiatric rehabilitation group
	Target Number of Participant	100
	Arm Type	Experimental
	Arm Description	1. Physical activity and Breathing Exercises<br>1) Physical activity<br>- Stretching and strengthening exercise<br>- Starting with the joint range of motion, gradually progresses with muscle strengthening movement.<br><br>2) Breathing Exercises and Stabilization Techniques<br>: Exercise is performed twice a day during hospitalization in the following order. In the morning, exercise with the researcher(15 minutes), and the patient do exercise on their own at the afternoon(15 minutes).<br><br>(1) Diaphragmatic breathing (3 minutes)<br>① In a lying position, place one hand on your chest and the other on your stomach.<br>② Take a deep breath through your nose to inflate your stomach. At this time, make sure that you do not move your chest as much as possible with your hand on top of your chest.<br>③ Close your mouth and exhale slowly. At this time, the chest should not move as much as possible, and only the movement of the stomach should appear.<br><br>(2) Pursed-lip breathing (3 minutes)<br>① Relax your neck and shoulder muscles.<br>② Inhalation: Inhale slowly through the nose for 2 counts<br>③ Exhalation: Make the shape of your lips like a whistle and exhale slowly for 4 counts<br><br>(3) Square box breathing (3 minutes)<br>① Inhale: Close your eyes. Breathe in through your nose whilst counting to 4.<br>② Hold: Keep the air inside and count to 4.<br>③ Exhale: Breathe out slowly for count of 4. Repeat.<br>④ Hold:Wait another count of 4 until you breathe in through your nose again.<br><br>(4) huff coughing (3 minutes)<br>① Take a seated position.<br>② Breathe in through your mouth. At this point, breathe in a little deeper than usual.<br>③ Activate the abdominal muscles to quickly exhale and exhale 3 times with 'ha', 'ha', and 'ha' sounds.<br><br>(5) Progressive muscle relaxation (3 minutes)<br>① Get into a comfortable position.<br>② Take a few deep breaths and tense up the muscles.

		③ Exhale and release tension.<br>Upper extremity, shoulder, head, neck, chest, abdomen, lower extremities in or der<br><br>2. Occupational activity (Craft activity)<br>- Knitting, curling book, figure making, masterpiece puzzle, etc.<br>- After selecting the activity, the patient wants through a phone interview, the t herapist visits and provides it<br>- Educate yourself so that you can do it yourself and check if it was implemente d over the phone<br><br>3. Medical and conservative treatment<br>- Isolation and conservative treatment according to COVID-19 response guidelin es<br>- Implemented according to the patient's condition by medical staff
Arm 2	Arm Lab el	Coventional treatment group
	Target N umber o f Partici pant	100
	Arm Typ e	No intervention
	Arm De scription	1. Medical and conservative treatment<br>- Isolation and conservative treatment according to COVID-19 response guidelin es<br>- Implemented according to the patient's condition by medical staff

9. Subject Eligibility

Condition(s)/Problem (s)		* (U00-U99)Codes for special purposes<br>(U07.1)Coronavirus disease 2019, virus identified [COVID-19, virus identified]<br><br>COVID-19
Rare Disease		No
Inclusio n Criteria	Gender	Both
	Age	18Year~No Limit
	Descript ion	1. Diagnosed with COVID-19 in positive finindgs of PCR test<br>2. Classified as mild by medical staff<br>: Oxygen saturation is lower than 95% or during oxygen therapy<br>3. Isolated in hosipital<br>4. No history of diagnosis of psychiatric disorders<br>5. No communication problems

	6. Adults 18 years of age or older<br>7. Understands the purpose of the research and voluntarily agrees.
Exclusion Criteria	1. Not medically stable<br>2. Have difficulty of using Korean<br>3. Have a history of diagnosis of psychiatric disorders
Healthy Volunteers	Yes

10. Outcome Measure(s)

Type of Primary Outcome	Not applicable

- Primary Outcome(s) 1

Outcome	SAS(self rating anxiety scale)
Timepoint	Before intervention once, after intervention once.

- Primary Outcome(s) 2

Outcome	SDS(self rating depression scale)
Timepoint	Before intervention once, after intervention once.

- Primary Outcome(s) 3

Outcome	PHQ-9(Patient Health Questionnaire-9)
Timepoint	Before intervention once, after intervention once.

- Secondary Outcome(s) 1

Outcome	VAS(Visual Analogue Scale)
Timepoint	Before intervention once, after intervention once.

- Secondary Outcome(s) 2

Outcome	ISI(Insomnia severity index)
Timepoint	Before intervention once, after intervention once.

11. Study Results and Publication

Result Registered	No

12. Sharing of Study Data(Deidentified Individual-Patient Data, IPD)

Sharing Statement	No

목록으로 이동
