# Supplementary material for: Psychological rehabilitation for isolated patients with COVID-19 infection: A randomized controlled study
Source: PLoS One. 2022 Dec 27;17(12):e0278475. doi: 10.1371/journal.pone.0278475 (PMC9794049; doi:10.1371/journal.pone.0278475)
Supplement: S2 File — (PDF) [file pone.0278475.s003.pdf]

Model Information	
Data Set	WORK.JUNG_DATA
Dependent Variable	VAS_AN
Covariance Structure	Unstructured
Subject Effect	Code
Estimation Method	REML
Residual Variance Method	None
Fixed Effects SE Method	Model-Based
Degrees of Freedom Method	Between-Within

Class Level Information		
Class	Levels	Values
Code	109	1 2 3 4 5 6 7 9 10 11 13 14 15 16 18 19 20 21 22 23 24 25 27 28 29 30 31 34 35 36 37 38 39 40 41 44 45 46 47 49 53 55 56 58 59 60 65 66 70 71 72 73 75 76 81 83 84 85 86 87 90 91 92 93 94 95 96 97 101 102 103 104 105 106 108 110 112 114 115 116 117 118 122 126 127 128 129 131 134 135 136 139 140 141 143 144 145 146 147 148 149 151 152 154 155 157 158 159 160
Group	2	1 2
Time	2	1 2

Dimensions	
Covariance Parameters	3
Columns in X	9
Columns in Z	0
Subjects	109
Max Obs per Subject	2

Number of Observations	
Number of Observations Read	218
Number of Observations Used	218
Number of Observations Not Used	0

Iteration History			
Iteration	Evaluations	-2 Res Log Like	Criterion
0	1	1004.25659645	
1	1	979.76499635	0.00000000

Convergence criteria met.

Covariance Parameter Estimates		
Cov Parm	Subject	Estimate
UN(1,1)	Code	7.3448
UN(2,1)	Code	2.2799
UN(2,2)	Code	4.5172

Fit Statistics	
-2 Res Log Likelihood	979.8
AIC (Smaller is Better)	985.8
AICC (Smaller is Better)	985.9
BIC (Smaller is Better)	993.8

Null Model Likelihood Ratio Test

DF	Chi-Square	Pr > ChiSq
2	24.49	<.0001

Type 3 Tests of Fixed Effects

Effect	Num DF	Den DF	F Value	Pr > F
Group	1	107	0.50	0.4818
Time	1	107	24.29	<.0001
Group*Time	1	107	10.47	0.0016

Model Information	
Data Set	WORK.JUNG_DATA
Dependent Variable	SAS
Covariance Structure	Unstructured
Subject Effect	Code
Estimation Method	REML
Residual Variance Method	None
Fixed Effects SE Method	Model-Based
Degrees of Freedom Method	Between-Within

Class Level Information

Class	Levels	Values
Code	109	1 2 3 4 5 6 7 9 10 11 13 14 15 16 18 19 20 21 22 23 24 25 27 28 29 30 31 34 35 36 37 38 39 40 41 44 45 46 47 49 53 55 56 58 59 60 65 66 70 71 72 73 75 76 81 83 84 85 86 87 90 91 92 93 94 95 96 97 101 102 103 104 105 106 108 110 112 114 115 116 117 118 122 126 127 128 129 131 134 135 136 139 140 141 143 144 145 146 147 148 149 151 152 154 155 157 158 159 160
Group	2	1 2
Time	2	1 2

Dimensions	
Covariance Parameters	3
Columns in X	9
Columns in Z	0
Subjects	109
Max Obs per Subject	2

Number of Observations	
Number of Observations Read	218
Number of Observations Used	218
Number of Observations Not Used	0

Iteration History

Iteration	Evaluations	-2 Res Log Like	Criterion
0	1	1449.54283822	
1	1	1396.51483349	0.00000000

Convergence criteria met.

Covariance Parameter Estimates

Cov Parm	Subject	Estimate
UN(1,1)	Code	59.5930
UN(2,1)	Code	27.1328
UN(2,2)	Code	35.4303

Fit Statistics	
-2 Res Log Likelihood	1396.5
AIC (Smaller is Better)	1402.5
AICC (Smaller is Better)	1402.6
BIC (Smaller is Better)	1410.6

Null Model Likelihood Ratio Test

DF	Chi-Square	Pr > ChiSq
2	53.03	<.0001

Type 3 Tests of Fixed Effects

Effect	Num DF	Den DF	F Value	Pr > F
Group	1	107	3.08	0.0822
Time	1	107	45.04	<.0001
Group*Time	1	107	12.46	0.0006

Model Information	
Data Set	WORK.JUNG_DATA
Dependent Variable	VAS_DE
Covariance Structure	Unstructured
Subject Effect	Code
Estimation Method	REML
Residual Variance Method	None

Model Information	
Fixed Effects SE Method	Model-Based
Degrees of Freedom Method	Between-Within

Class Level Information		
Class	Levels	Values
Code	109	1 2 3 4 5 6 7 9 10 11 13 14 15 16 18 19 20 21 22 23 24 25 27 28 29 30 31 34 35 36 37 38 39 40 41 44 45 46 47 49 53 55 56 58 59 60 65 66 70 71 72 73 75 76 81 83 84 85 86 87 90 91 92 93 94 95 96 97 101 102 103 104 105 106 108 110 112 114 115 116 117 118 122 126 127 128 129 131 134 135 136 139 140 141 143 144 145 146 147 148 149 151 152 154 155 157 158 159 160
Group	2	1 2
Time	2	1 2

Dimensions	
Covariance Parameters	3
Columns in X	9
Columns in Z	0
Subjects	109
Max Obs per Subject	2

Number of Observations	
Number of Observations Read	218
Number of Observations Used	218
Number of Observations Not Used	0

Iteration History			
Iteration	Evaluations	-2 Res Log Like	Criterion
0	1	990.45880752	
1	1	955.32756172	0.00000000

Convergence criteria met.

Covariance Parameter Estimates		
Cov Parm	Subject	Estimate
UN(1,1)	Code	7.2389
UN(2,1)	Code	2.4161
UN(2,2)	Code	3.8825

Fit Statistics	
-2 Res Log Likelihood	955.3
AIC (Smaller is Better)	961.3
AICC (Smaller is Better)	961.4
BIC (Smaller is Better)	969.4

Null Model Likelihood Ratio Test		
DF	Chi-Square	Pr > ChiSq
2	35.13	<.0001

Type 3 Tests of Fixed Effects				
Effect	Num DF	Den DF	F Value	Pr > F
Group	1	107	0.04	0.8429
Time	1	107	16.30	0.0001
Group*Time	1	107	23.42	<.0001

Model Information	
Data Set	WORK.JUNG_DATA
Dependent Variable	SDS
Covariance Structure	Unstructured
Subject Effect	Code
Estimation Method	REML
Residual Variance Method	None
Fixed Effects SE Method	Model-Based
Degrees of Freedom Method	Between-Within

Class Level Information		
Class	Levels	Values
Code	109	1 2 3 4 5 6 7 9 10 11 13 14 15 16 18 19 20 21 22 23 24 25 27 28 29 30 31 34 35 36 37 38 39 40 41 44 45 46 47 49 53 55 56 58 59 60 65 66 70 71 72 73 75 76 81 83 84 85 86 87 90 91 92 93 94 95 96 97 101 102 103 104 105 106 108 110 112 114 115 116 117 118 122 126 127 128 129 131 134 135 136 139 140 141 143 144 145 146 147 148 149 151 152 154 155 157 158 159 160
Group	2	1 2
Time	2	1 2

Dimensions	
Covariance Parameters	3
Columns in X	9
Columns in Z	0
Subjects	109
Max Obs per Subject	2

Number of Observations	
Number of Observations Read	218
Number of Observations Used	218
Number of Observations Not Used	0

Iteration History			
Iteration	Evaluations	-2 Res Log Like	Criterion
0	1	1568.73369673	
1	1	1513.90956348	0.00000000

Convergence criteria met.

Covariance Parameter Estimates		
Cov Parm	Subject	Estimate
UN(1,1)	Code	88.1163
UN(2,1)	Code	52.2503
UN(2,2)	Code	77.7344

Fit Statistics	
-2 Res Log Likelihood	1513.9
AIC (Smaller is Better)	1519.9
AICC (Smaller is Better)	1520.0
BIC (Smaller is Better)	1528.0

Null Model Likelihood Ratio Test		
DF	Chi-Square	Pr > ChiSq
2	54.82	<.0001

Type 3 Tests of Fixed Effects				
Effect	Num DF	Den DF	F Value	Pr > F
Group	1	107	0.23	0.6348
Time	1	107	25.30	<.0001
Group*Time	1	107	6.76	0.0106

Model Information	
Data Set	WORK.JUNG_DATA
Dependent Variable	PHQ9
Covariance Structure	Unstructured
Subject Effect	Code
Estimation Method	REML
Residual Variance Method	None
Fixed Effects SE Method	Model-Based
Degrees of Freedom Method	Between-Within

Class Level Information		
Class	Levels	Values
Code	109	1 2 3 4 5 6 7 9 10 11 13 14 15 16 18 19 20 21 22 23 24 25 27 28 29 30 31 34 35 36 37 38 39 40 41 44 45 46 47 49 53 55 56 58 59 60 65 66 70 71 72 73 75 76 81 83 84 85 86 87 90 91 92 93 94 95 96 97 101 102 103 104 105 106 108 110 112 114 115 116 117 118 122 126 127 128 129 131 134 135 136 139 140 141 143 144 145 146 147 148 149 151 152 154 155 157 158 159 160
Group	2	1 2
Time	2	1 2

Dimensions	
Covariance Parameters	3
Columns in X	9
Columns in Z	0
Subjects	109
Max Obs per Subject	2

Number of Observations	
Number of Observations Read	218
Number of Observations Used	218
Number of Observations Not Used	0

Iteration History			
Iteration	Evaluations	-2 Res Log Like	Criterion
0	1	1282.17629670	
1	1	1211.38625526	0.00000000

Convergence criteria met.

Covariance Parameter Estimates		
Cov Parm	Subject	Estimate
UN(1,1)	Code	28.9276
UN(2,1)	Code	13.2993
UN(2,2)	Code	14.5408

Fit Statistics	
-2 Res Log Likelihood	1211.4
AIC (Smaller is Better)	1217.4
AICC (Smaller is Better)	1217.5
BIC (Smaller is Better)	1225.5

Null Model Likelihood Ratio Test		
DF	Chi-Square	Pr > ChiSq
2	70.79	<.0001

Type 3 Tests of Fixed Effects				
Effect	Num DF	Den DF	F Value	Pr > F
Group	1	107	1.48	0.2265
Time	1	107	8.58	0.0041
Group*Time	1	107	8.58	0.0041

Model Information	
Data Set	WORK.JUNG_DATA
Dependent Variable	ISI
Covariance Structure	Unstructured
Subject Effect	Code
Estimation Method	REML
Residual Variance Method	None
Fixed Effects SE Method	Model-Based
Degrees of Freedom Method	Between-Within

Class Level Information		
Class	Levels	Values
Code	109	1 2 3 4 5 6 7 9 10 11 13 14 15 16 18 19 20 21 22 23 24 25 27 28 29 30 31 34 35 36 37 38 39 40 41 44 45 46 47 49 53 55 56 58 59 60 65 66 70 71 72 73 75 76 81 83 84 85 86 87 90 91 92 93 94 95 96 97 101 102 103 104 105 106 108 110 112 114 115 116 117 118 122 126 127 128 129 131 134 135 136 139 140 141 143 144 145 146 147 148 149 151 152 154 155 157 158 159 160
Group	2	1 2
Time	2	1 2

Dimensions	
Covariance Parameters	3
Columns in X	9
Columns in Z	0
Subjects	109
Max Obs per Subject	2

Number of Observations	
Number of Observations Read	218
Number of Observations Used	218
Number of Observations Not Used	0

Iteration History			
Iteration	Evaluations	-2 Res Log Like	Criterion
0	1	1367.24875907	
1	1	1278.53780845	0.00000000

Convergence criteria met.

Covariance Parameter Estimates		
Cov Parm	Subject	Estimate
UN(1,1)	Code	45.7310
UN(2,1)	Code	20.2564
UN(2,2)	Code	18.9566

Fit Statistics	
-2 Res Log Likelihood	1278.5
AIC (Smaller is Better)	1284.5
AICC (Smaller is Better)	1284.7
BIC (Smaller is Better)	1292.6

Null Model Likelihood Ratio Test		
DF	Chi-Square	Pr > ChiSq
2	88.71	<.0001

Type 3 Tests of Fixed Effects				
Effect	Num DF	Den DF	F Value	Pr > F
Group	1	107	0.18	0.6699
Time	1	107	14.25	0.0003
Group*Time	1	107	4.41	0.0381
